# Dataset for evaluation of threescoring systems for forecasting the clinical outcomes of patients with upper gastrointestinal bleeding (UGIB) – Ahvaz, Iran

**DOI:** 10.1016/j.dib.2018.10.029

**Published:** 2018-10-12

**Authors:** Seyed Saeidseyedian, Ali Akbar Shayesteh, Fereshteh Beyranvandi

**Affiliations:** a Alimentary Tract Research Center, Jundishapur University of Medical Science, Ahvaz, Iran; b Department of Gastroenterology, Research Center for Infectious Diseases of Digestive System, Ahvaz Jundishapur University of Medical Sciences, Ahvaz, Iran

**Keywords:** Rockall scoring system, Blatchford scoring system, Upper gastrointestinal bleeding, Ahvaz

## Abstract

Upper gastrointestinal bleeding (UGIB) which occurs proximal to the Treitz ligament is one of the most common cases is emergency medical conditions. The aim of this data article is to evaluation of Rockall and Blatchford scoring systems for predicting the clinical outcomes of patients with upper gastrointestinal bleeding in Imam-Khomeini Hospital, Ahvaz, Iran. This dataset was collected by retrospective descriptive epidemiologic survey which 350 non-cirrhotic patients with UGIB who referred to Ahwaz Imam-Khomeini Hospital for six months. According to the obtained data, in both clinical Rockall and complete Rockall systems, the need for re-endoscopy and the risk of re-bleeding in patients with high scores was more compared to patients with low scores. While, the obtained data about Blatchford score for re-endoscopy and re-bleeding risk was showed which no significant difference. Based on to present dataset, the Rockall systems was superior to Blatchford systems in predicting the re-bleeding as well as the need for re-endoscopy while, none of the systems were efficient in terms of predicting the need for urgent endoscopy and surgery.

**Specifications table**

**Table:** 

Subject area	Medicine
More specific subject area	Emergency medicine
Type of data	Tables and figures
How data was acquired	This dataset was collected by retrospective descriptive epidemiologic survey which 350 non-cirrhotic patients with UGIB who referred to Ahwaz Imam-Khomeini Hospital for six months.
Data format	Raw, analyzed
Experimental factors	Required data were extracted through a questionnaire. The questionnaire included demographic data, clinical symptoms, endoscopic findings, and clinical outcomes.
Experimental features	The age of over 18 years was regarded as inclusion criterion, while the patients with cirrhosis were excluded from this survey.
Data source location	Ahvaz city, Iran
Data accessibility	Data were included in this article
Related research article	D. Dicu, F. Pop, D. Ionescu, T. Dicu, Comparison of risk scoring systems in predicting clinical outcome at upper gastrointestinal bleeding patients in an emergency unit, Am. J. Emerg. Med. 31 (2013) 94-9 [1]. I.C.Chen, M.S. Hung, T.F. Chiu, J.C. Chen, C.T.Hsiao, Risk scoring systems to predict need for clinical intervention for patients with nonvariceal upper gastrointestinal tract bleeding, Am. J. Emerg. Med. 25 (2007)774-9 [2].

**Value of the data**

• To conduct further research on the topic under discussion, the obtained data from this dataset can be the basis for performance of the future similar studies.
• The acquired data from present dataset can be useful to decide about selection of the most effective scoring systems for predicting the clinical outcomes of patients with upper gastrointestinal bleeding.
• The obtained data from this dataset can be useful to increase patient satisfaction as well as reduce the cost of the health system.

## 1 Data

According to the demographic data, the patients were 18–94 years old, with the standard deviation of 20.63. Out of 350 patients studied, 230 patients were males (65.7%) and 120 patients were female (34.3%).

**Fig. 1:**
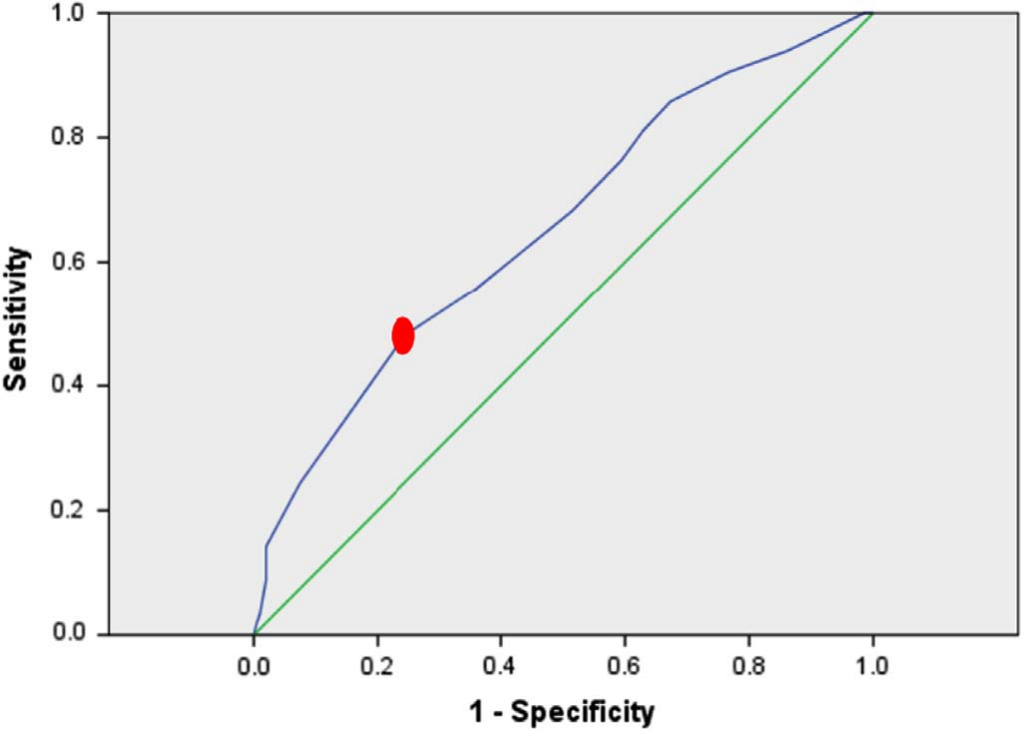
The characteristic curve for the Blatchford scoring system.

**Fig. 2:**
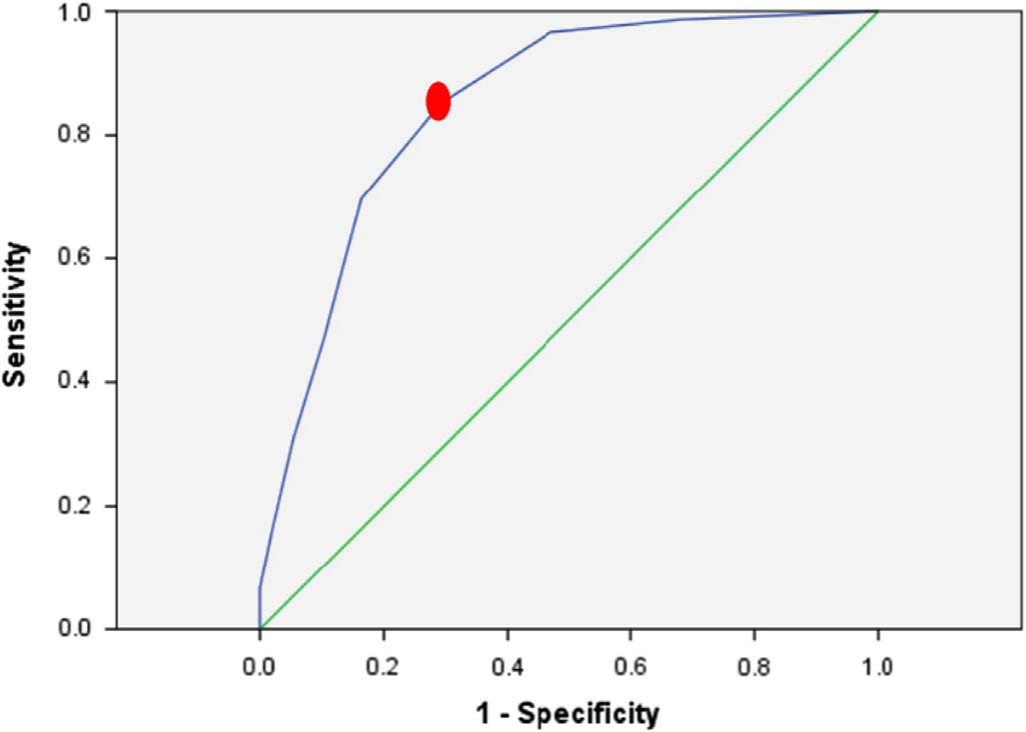
The characteristic curve for clinical Rockall system.

**Fig. 3:**
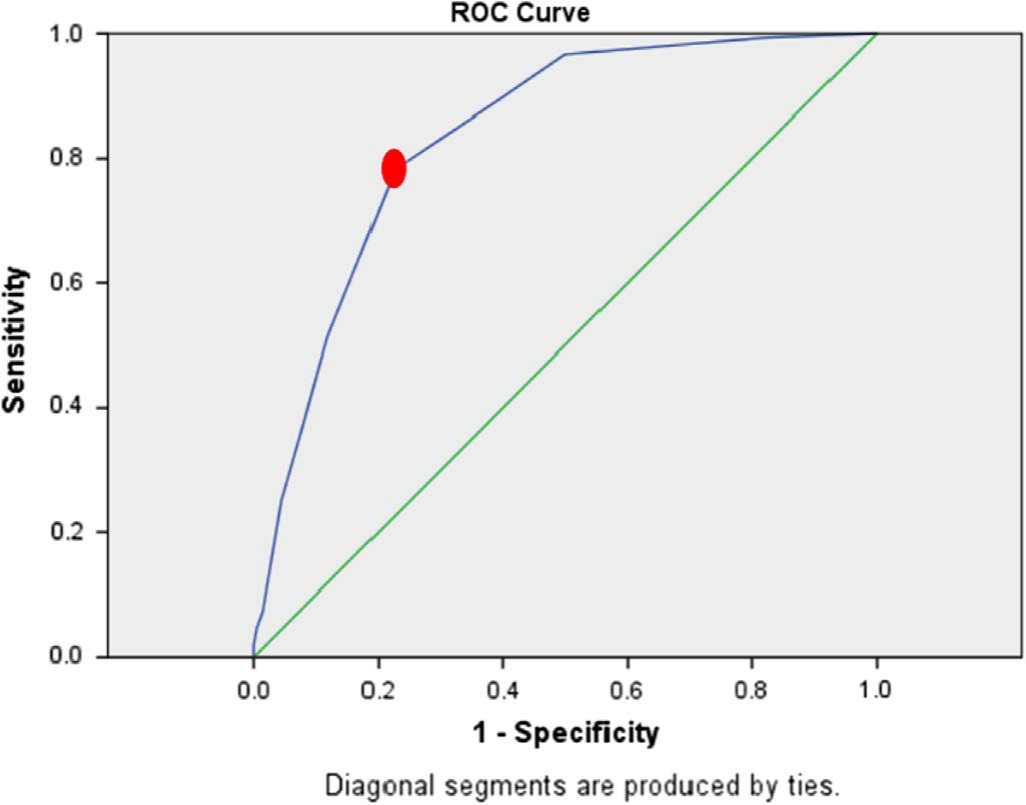
The characteristic curve for the complete Rockall system.

Among the patients, 126 (36%) patients suffered from hemostasis, 124 (35.42%) from melena, and 88 patients (25.14%) from both hemostasis and melena. Moreover, 4 patients (1.4%) complained about syncope, and 9 patients (2.57%) complained both about syncope and melena which were the most common causes of the referral of patients with gastrointestinal hematemesis. The frequency of clinical findings related to selected patients were shown in Table 1. The classification of the patients into low-risk and high-risk groups based on mean value (cut-off) for the three scoring systems were shown in Table 2. In addition, the characteristic curve for the Blatchford, clinical Rockall and complete Rockall scoring systems were provided in Fig. 1, Fig. 2, Fig. 3, respectively. Table 3 shows the relationship between clinical parameters and scoring systems. In Table 4, the clinical outcomes of patients was divided into low-risk and high-risk groups for three scoring systems.

**Table 1:** The frequency of clinical findings.

**Outcomes**	**Frequency**	
	**Number**	**Percent**
Re-bleeding	38	10.85
Re-endoscopy	50	14. 3
Surgery	3	0.9
Mortality	26	9.15
Transfusion	207	59.14
Urgent endoscopy	163	46.57
No endoscopy	24	6.8

**Table 2:** Classification of the patients into low-risk and high-risk groups based on mean value (cut-off).

**Scoring systems**	**Cut off**	**Sensitivity**	**Specificity**	**Low Risk**	**High Risk**	**Total**
Blatchford	11.5	55.4	64.4	196	154	350
Clinical Rockall	2.5	79.7	80.2	192	158	350
Complete Rockall	3.5	78.4	77.2	188	162	350

**Table 3:** The relationship between clinical parameters and scoring systems.

**Clinical parameters**	**Blatchford**	**Clinical Rockall**	**Complete Rockall**
Re-endoscopy	0.35^a^	0.02^a^	0.001^a^
Re-bleeding	0.25	0.01	0.001
Surgery	0.08	0.59	0.09
Transfusion	0.001	0.001	0.001
Mortality	0.001	0.001	0.001
Urgent endoscopy	0.07	0.33	0.39

a Correlation coefficient.

**Table 4:** Dividing the clinical outcomes of patients into low-risk and high-risk groups for three scoring systems.

**Scoring system**	**Risk level**	**Re-endoscopy**	**Re-bleeding**	**Surgery**	**Transfusion**	**Mortality**	**Urgent endoscopy**
Blatchford	Low risk: 196	25 (12.8%)	18 (9.2%)	0 (0%)	69 (35.2%)	4 (2%)	79 (40.3%)
	High risk: 154	25 (16.2%)	20 (13%)	(1.9%) 3	138 (89.6%)	22 (14.3%)	48 (31.2%)
Clinical Rockall	Low risk: 192	20 (10.4%)	14 (7.3%)	1 (0.5%)	86 (44.8%)	3 (1.6%)	74 (38.5%)
	High risk: 158	30 (19%)	24 (15.2%)	2 (1.3%)	121 (76.6%)	(14.6%) 23	53 (33.5%)
Complete Rockall	Low risk: 188	16 (8.5%)	9 (4.8%)	0 (0%)	81 (43.1%)	5 (2.7%)	72 (38.3%)
	High risk: 162	34 (21%)	29 (17.9%)	3 (1.9%)	126 (77.8%)	21 (13%)	55 (34%)

## 2 Experimental design, materials and methods

The aim of the data article is to evaluate the three scoring systems including clinical Rockall, Complete Rockall and Blatchford for forecasting the clinical outcomes of patients with upper gastrointestinal bleeding (UGIB) This dataset was collected by retrospective descriptive epidemiologic survey which 350 non-cirrhotic patients with UGIB who referred to Ahwaz Imam-Khomeini Hospital for six months. The medical records and required data were extracted through a questionnaire. The questionnaire included demographic data, clinical symptoms, endoscopic findings, and clinical outcomes [1], [2]. In addition, the outcomes included re-bleeding, the need for re-endoscopy, surgery and patient mortality. The need for blood transfusion or endoscopy was recorded as an emergency in the questionnaire. After Blatchford score were inserted, the clinical Rockall and complete Rockall scored were determined for each patient. The age of over 18 years was regarded as inclusion criterion, while the patients with cirrhosis were excluded from the study. According to Blatchford, clinical Rockall, and complete Rockall scoring systems, patients were evaluated and scored. In order to classify the patients into two low-risk and high-risk groups based on the scoring systems mentioned above, the statistical characteristic curve (ROC) was used which was defined as a cut-off with specific sensitivity and specificity. In order to determine the relationship between the variables and the clinical outcomes, the Chi Square test was used by SPSS.21 software (SPSS Inc. Chicago, IL, USA).
